# *PRDM16* expression is an independent prognostic factor in AML with the double-mutant *NPM1*/*FLT3*-ITD genotype

**DOI:** 10.1007/s00277-026-06767-x

**Published:** 2026-01-22

**Authors:** Sebastian Stasik, Jan-Niklas Eckardt, Christoph Röllig, Claudia D. Baldus, Hubert Serve, Carsten Müller-Tidow, Kerstin Schäfer-Eckart, Martin Kaufmann, Stefan W. Krause, Mathias Hänel, Andreas Neubauer, Gerhard Ehninger, Uwe Platzbecker, Martin Bornhäuser, Johannes Schetelig, Jan M. Middeke, Christian Thiede

**Affiliations:** 1 Medizinische Klinik und Poliklinik I, Medizinische Fakultät Carl Gustav Carus, Technische Universität Dresden, Dresden, Germany; 2 Klinik Für Innere Medizin II, Universitätsklinikum Schleswig-Holstein, Hämatologie Und Onkologie, Kiel, Germany; 3 Medizinische Klinik II, Universitätsklinikum Frankfurt, Frankfurt Am Main, Germany; 4 Medizinische Klinik V, Universitätsklinikum Heidelberg, Heidelberg, Germany; 5 Klinik Für Innere Medizin V, Klinikum Nürnberg Nord, Nuremberg, Germany; 6 Abteilung Für Hämatologie, Onkologie Und Palliativmedizin, Robert-Bosch-Krankenhaus, Stuttgart, Germany; 7 Medizinische Klinik 5, Universitätsklinikum Erlangen, Erlangen, Germany; 8 Medizinische Klinik III, Klinikum Chemnitz, Chemnitz, Germany; 9 Klinik Für Hämatologie, Philipps Universität Marburg, Onkologie, ImmunologieMarburg, Germany; 10 National Center for Tumor Diseases, Dresden, Germany; 11 DKMS Clinical Trials Unit, Dresden, Germany; 12 AgenDix GmbH, Dresden, Germany

**Keywords:** *PRDM16* expression, Acute Myeloid Leukemia (AML), Clinical Outcome, Molecular associations

## Abstract

PRDM16 (PR Domain Containing 16) is a transcription factor that plays a critical role in hematopoietic stem cell maintenance. In acute myeloid leukemia (AML), *PRDM16* overexpression is linked to specific cytogenetic risk groups and poor prognosis. However, in *NPM1*-mutated AMLs, *PRDM16* expression varies widely, with no consensus on its prognostic significance. To understand molecular and clinical associations of *PRDM16* expression in this relevant subgroup, we screened 503 adult *NPM1*-mutant AML patients. High *PRDM16* expression was associated with mutations in *DNMT3A* (57% vs 22%; *p* < 0.0001) and *FLT3*-ITD (51% vs 37%; *p* = 0.0258), and therefore a higher rate of ELN2022 intermediate-risk (42% vs 26%; *p* = 0.01), compared to low *PRDM16* expression. Accordingly, *PRDM16* overexpression was not associated with clinical outcome in multivariable analysis adjusting for ELN2022 risk in the unselected *NPM1*-mutant AML cohort. However, within the double-mutant *NPM1*/*FLT3*-ITD subgroup (n = 200), low *PRDM16* expression was an independent prognostic factor for longer survival (hazard ratio [95%-CI] 0.467 [0.270–0.807]; *p* = 0.006). On a molecular level, low *PRDM16* expression was associated with mutations in epigenetic regulators (*TET2*, *IDH1*/*2*) and increased *PRDM16* promoter methylation, suggesting impaired TET/IDH-mediated DNA-demethylation as underlying mechanism. Notably, *IDH1* R132C and *IDH2* R140Q alterations particularly contributed to higher *PRDM16* promoter methylation and reduced expression. These results suggest an association of *PRDM16* overexpression with the *NPM1*/*FLT3*-ITD/*DNMT3A* triple-mutant AML genotype, typically linked to high leukemia stem cell frequencies and poor prognosis. Importantly, within this adverse AML subtype low *PRDM16* expression is an independent prognostic marker for favorable outcome, supporting an anti-leukemic mechanism in AMLs with repressed *PRDM16* transcription.

**Graphical Abstract:**

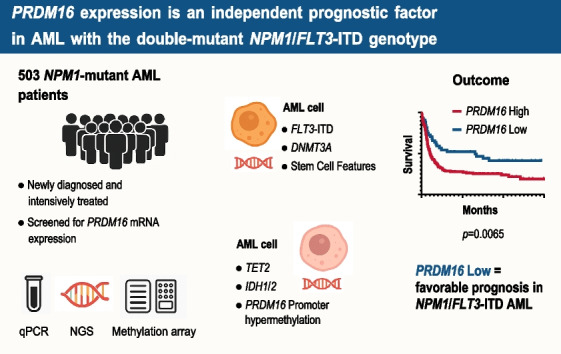

**Supplementary Information:**

The online version contains supplementary material available at 10.1007/s00277-026-06767-x.

## Introduction

Acute myeloid leukemia (AML) is a group of myeloid stem cell malignancies characterized by expansion of aberrant early hematopoietic stem and progenitor cells [1]. Although research over the last decades have advanced our understanding of disease mechanisms and led to some improvements in treatment options and outcome, prognosis of the majority of patients is still dismal and the majority of affected individuals will finally succumb to the disease [2]. One major mechanism of resistance is the inability to eradicate leukemic stem cells, which ultimately leads to relapse [3]. Improving our knowledge on these leukemic stem cells and what discerns them from their normal counterparts thus is of critical importance to further improve outcome. One of the proteins recently associated with leukemic stem cells is PRDM16 [4–6].

The *PRDM16* gene, located on chromosome 1p36, encodes a protein that contains a PR domain and two zinc finger motifs, which are essential for DNA binding and its function in transcriptional regulation [7, 8]. As a transcription factor, PRDM16 plays a critical role in regulating various biological processes, particularly cell differentiation, metabolism, and stress responses [7].

Initially recognized for its role in brown adipose tissue differentiation, PRDM16 regulates hematopoietic and neural stem cell maintenance, primarily through protein–protein interactions with key transcription factors and chromatin-modifying complexes [5–9]. In human pathology, molecular alterations (deletions and point mutations) of the *PRDM16* gene are associated with cardiomyopathy [10], while chromosomal rearrangements involving *PRDM16* (such as translocations with *RUNX1* or *ETV6*), contribute to hematological malignancies like AML and myelodysplastic neoplasms (MDS), often leading to poor prognosis [11–14].

Furthermore, recent studies have highlighted the significance of *PRDM16* overexpression [15–18] and a differential role of *PRDM16* isoforms in the pathogenesis of AML [19–22]. More specifically, increased expression of *PRDM16*, particularly of the short isoform (lacking the PR-domain), has been associated with adverse prognostic subgroups like *FLT3*-ITD, *KMT2A*-PTD, and *NUP98*-*NSD1*, and poor outcome [15–18, 23]. Biologically, *PRDM16* overexpression in AML cells may promote leukemic cells to survive under stress conditions of the bone marrow microenvironment, contributing to their resistance to differentiation, cell death and treatment [5, 9]. Vice versa, down-regulation of *PRDM16* expression was observed in clinically favorable-risk AMLs, such as t(8;21) and inv(16) [15, 17, 23, 24] and associated with a specific antileukemic mechanism by which expanding hematopoietic stem cells (HSCs) avoid leukemic transformation [25]. Therefore, *PRDM16* expression levels may serve as a prognostic marker in AML, potentially refining risk-stratification of AML patients and guiding treatment decisions.

However, while *PRDM16* expression in AML has been associated with varied prognoses depending on specific cytogenetic risk groups, *PRDM16* expression shows considerable variability within other AML subtypes, particularly in cases with a normal karyotype [18, 23]. Also in *NPM1*-mutated AML, which accounts for approximately 30% of all AML cases [26], *PRDM16* expression varies widely, with no clear consensus on its prognostic significance [15, 23]. Furthermore, the prognostic implication of *PRDM16* expression have primarily been investigated in smaller cohorts of adult AML patients (n = 121, 151, 267) [15–17] or in pediatric AML [18, 23].

We recently performed RNA sequencing in several molecularly well-defined AML subgroups [27] and saw a clear differentiation of low (e.g. CBF-leukemias, *CEBPA*-bZIP-Indel) and high *PRDM16* expression (*NUP98*-*NSD1*; *UBTF*-TD). However, in *NPM1*-mutant AML, this separation was less distinct, with both, low and high *PRDM16* expressing samples. Given the prognostic heterogeneity of *NPM1*-mutations, we wanted to investigate the impact of *PRDM16* expression in a large cohort of 503 intensively treated, well-annotated adult AML patients with *NPM1* mutations. Molecular and clinical associations were analyzed according to the *PRDM16* expression level. To better understand the epigenetic mechanisms underlying the pathogenesis in these AMLs, we explored the relationship of *PRDM16* promoter methylation profiles with molecular data and *PRDM16* expression levels.

## Methods

### Patients

We retrospectively screened 503 adult patients with *NPM1*-mutant AML. Eligibility criteria included newly diagnosed AML according to WHO definitions, age ≥ 18 years, and available biomaterial at diagnosis. Written informed consent was obtained, and the study was approved by the ethics board of the Technical University Dresden (EK98032010), following the Helsinki Declaration. Protocols were registered with the Study Alliance Leukemia (SAL) under NCT numbers 00180115 (AML96), 00180102 (AML2003), 00180167 (AML60 +), and 00893373 (SORAML). All protocols involved intensive induction chemotherapy and consolidation treatment. None of the patients received FLT3 inhibitors or other targeted agents, ensuring a homogeneous treatment backbone across the entire cohort.

### Quantitative RT-PCR analysis for PRDM16 expression

Total RNA was isolated from bone marrow or peripheral blood at diagnosis using the RNeasy Mini Kit (Qiagen) on a QIAcube (Qiagen). cDNA conversion was performed with the SuperScript VILO MasterMix, using 11.5 µL of RNA for reverse transcription (Invitrogen). Quantitative RT-PCR for total *PRDM16* mRNA (*tPRDM16*) was conducted using a TaqMan Gene Expression Assay (Hs00922674_ml; exon 14/15 junction; Thermo Fisher Scientific) and the TaqMan Universal Master Mix on a 7500 qRT-PCR device (Applied Biosystems). 1 µL of cDNA was used per PCR reaction, with cycling conditions: 50 °C for 2 min, initial denaturation at 95 °C for 10 min, and 45 cycles of 95 °C for 15 s and 60 °C for 1 min. *PRDM16* expression was normalized to *GAPDH* (Hs02786624_g1; Thermo Fisher Scientific) using Delta-CT values. *PRDM16* copy numbers were determined by linear regression of a serial dilution of a sample with known *PRDM16* copy number obtained from RNA-sequencing. To measure *PRDM16* isoforms copy numbers, a full-length *PRDM16* assay (*fPRDM16*; exon 2/3 junction) was performed, and the short isoform (*sPRDM16*) copy number was calculated as *sPRDM16* = *tPRDM16*—*fPRDM16*, as described previously [18].

### Molecular analysis

Genomic DNA was isolated from bone marrow or peripheral blood using the DNeasy Blood and Tissue Kit (Qiagen, Hilden, Germany) and quantified via NanoDrop spectrophotometer. Genomic alterations were profiled using targeted resequencing with the TruSight Myeloid Panel (Illumina), covering 54 genes recurrently mutated in AML, according to the manufacturer's guidelines. Sequencing was performed on a NextSeq (150 bp) or MiSeq (300 bp) NGS instrument (Illumina). Alignment of demultiplexed FastQ files, variant calling, and filtering were performed using the Sequence Pilot software package (JSI Medical Systems) with default settings and a 5% variant allele frequency (VAF) cutoff. Human genome build HG19 was used for mapping.

### PRDM16 promoter methylation

*PRDM16* promoter methylation profiles of 74 AML samples (*PRDM16*^Low^ n = 14; *PRDM16*^Int (Int−I+Int−II)^ n = 39; *PRDM16*^High^ n = 24) with sufficient biomaterial were analyzed using the Infinium MethylationEPIC BeadChip array (Illumina), according to the standard protocol. DNA methylation was quantified using 655 CG probes targeting the *PRDM16* promoter region and gene body (chromosome 1:2,984,445–3,354,467). CpG annotation in the *PRDM16* promoter was based on the Illumina manifest. Differentially methylated regions between the *PRDM16* expression subgroups were identified using PRISM version 10 (GraphPad Software).

### Statistical analysis

Variables between groups were compared using the Chi-squared or Mann–Whitney U test. For categorical variables with hierarchical order (ELN), the Cochrane-Armitage test for trend was used. Statistical significance was determined using a significance level α of 0.05. The odds ratio (OR) for complete remission (CR) after intensive induction therapy was evaluated using logistic regression models. Time-to-event variables including event-free survival (EFS), relapse-free survival (RFS), and overall survival (OS), were analyzed using Cox proportional hazard models to obtain hazard ratios (HR) as well as the Kaplan–Meier method and the log-rank test. For all OR and HR, 95%-confidence intervals (95%-CI) are reported. Analyses and visualizations were conducted in STATA BE 18.0 (Stata Corp) and PRISM v. 10 (GraphPad). For survival analyses, patients were first categorized into *PRDM16* expression quartiles (Q1–Q4) based on relative transcript levels. To improve statistical robustness in subgroup analyses, *PRDM16* High (Q4) and Intermediate (Q2–Q3) groups were also evaluated as a combined category (*PRDM16* High/Intermediate) due to overlapping survival curves, similar hazard ratios, and comparable molecular and epigenetic profiles distinct from *PRDM16* Low cases.

## Results

### PRDM16 expression levels

**Fig. 1:**
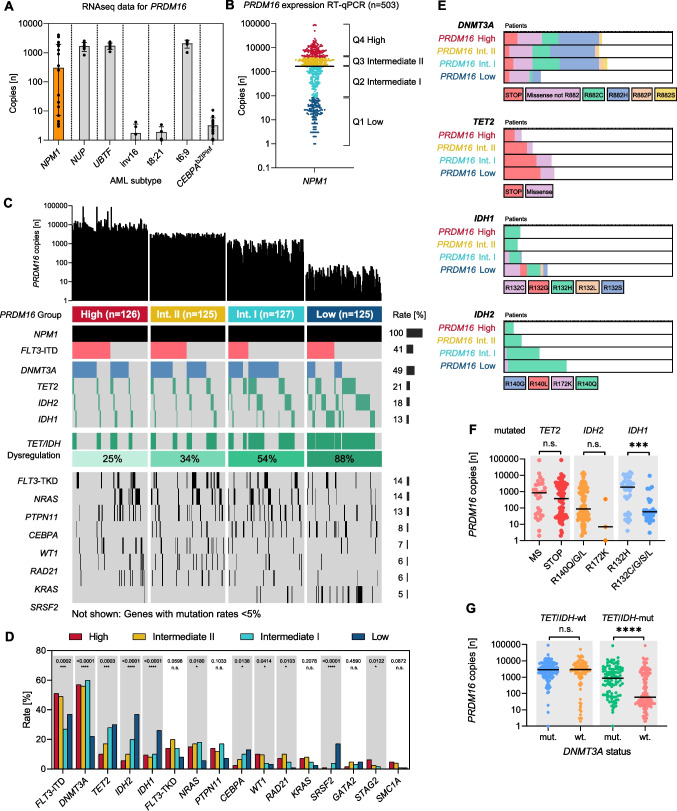
Molecular associations of *PRDM16* expression levels in *NPM1*-mutant AML. **(A)** Read counts (mean with standard deviation) of *PRDM16* mRNA in various molecular AML subtypes obtained from previously published RNA sequencing data [23]. **(B)** RT-qPCR based screening of *PRDM16* expression in 503 AML patients with *NPM1* mutation. Bar indicates median value. **(C)** Molecular characteristics of different *PRDM16* expression levels according to statistical quartiles: *PRDM16*^Low^, *PRDM16*^Int−I^, *PRDM16*^Int−II^, and *PRDM16*^High^. **(D)** Mutation rates of AML driver genes across *PRDM16* expression levels. **(E)** Relative contribution of specific mutations (coded by color) in epigenetic modifiers *DNMT3A*, *TET2*, *IDH1*, and *IDH2* across *PRDM16* expression levels. **(F)** Association of *TET*/*IDH* mutations with *PRDM16* mRNA copy numbers. **(F)** Interaction of *DNMT3A* –and *TET2*/*IDH1*/*2* mutational status with *PRDM16* expression. Bars indicate median values. Significant differences (*p* < 0.05) are flagged with stars (n.s. not significant)

An initial survey of published transcriptomic data [27] revealed high *PRDM16* expression variance in *NPM1*-mutant AML compared to other molecular AML subgroups (Fig. 1A). Clinically favorable-risk groups, such as AML with inv(16), t(8;21), and *CEBPA*^bZIPinf^, showed low *PRDM16* copy numbers, while AML with *NUP*, *UBTF*-TD and t(6;9) exhibited significantly higher *PRDM16* read counts. A subsequent qPCR based screening in 503 *NPM1*-mutant AML patients confirmed a broad range of *PRDM16* mRNA copy numbers: median 1692 (range, 1–89,845) (95%-CI, 1321–1948) (Fig. 1B). For dichotomization, samples were categorized into quartiles (Q1–Q4) based on statistical distribution of *PRDM16* expression levels: (mRNA copies median, range) *PRDM16*^Low^ (24, 1–83), *PRDM16*^Int−I^ (742, 85–1692), *PRDM16*^Int−II^ (2615, 1692–3780), and *PRDM16*^High^ (7143, 3806–89845).

### Laboratory and molecular associations

Descriptive statistics of clinical parameters and associated co-mutations revealed significant differences across *PRDM16* expression levels (Table 1). Absolute patient numbers and their distribution across *PRDM16* expression levels and molecular subgroups are provided in Supplementary Table S3. Although generally rare, a significantly higher prevalence of complex karyotype (4.3% vs 0–0.8%; *p* = 0.046) was found in the *PRDM16*^Low^ group. *PRDM16*^Low^ patients had a lower ELN2022 intermediate-risk frequency (26.1% vs 29.3–44.4%; *p* = 0.008), higher bone marrow (79% vs 69.5–70%; *p* = 0.0009) and peripheral blast counts (67% vs 20–56%; *p* = 0.0001), and lower platelet counts (48 × 10^9^/L vs 60–77.5 × 10^9^/L; *p* = 0.0018), compared to *PRDM16*^High^ and *PRDM16*^Int^. No differences were observed for patient’s age at diagnosis, AML type, the presence of a normal karyotype and other tested laboratory parameters (Table 1).

**Table 1:** Baseline patient characteristics with respect to *the PRDM16* expression status

Variable	*PRDM16*^Low^	*PRDM16*^Int−I^	*PRDM16*^Int−II^	*PRDM16*^High^	*p*
missing*	23/503 (4.6)				
n/N (%)	115/480 (24.0)	123/480 (25.6)	117/480 (24.4)	125/480 (26.0)	
Age (years), median (IQR)	58 (49–70)	58 (49–66)	55 (47–62)	54 (48–61)	0.514
Sex, n (%)					0.359
female	60 (52.2)	61 (49.6)	71 (60.7)	68 (54.4)	
male	55 (47.8)	62 (51.4)	46 (39.3)	57 (45.6)	
disease status, n (%)					
de novo	105 (91.3)	114 (92.7)	109 (93.2)	117 (93.6)	0.870
sAML	6 (5.2)	9 (7.3)	7 (6.0)	6 (4.8)	0.856
tAML	3 (2.6)	0	0	2 (1.6)	0.105
missing	1 (0.9)	0	1 (0.8)	0	
Normal karyotype, n (%)					0.279
Yes	78 (67.8)	98 (79.7)	85 (72.6)	103 (82.4)	
No	22 (19.1)	19 (15.4)	21 (17.9)	15 (12.0)	
missing	15 (13.0)	6 (4.9)	11 (9.4)	7 (5.6)	
Complex karyotype, n (%)					0.046
Yes	5 (4.3)	1 (0.8)	0	1 (0.8)	
No	93 (80.9)	104 (84.6)	98 (83.8)	109 (87.2)	
missing	17 (14.8)	18 (14.6)	19 (16.2)	15 (12.0)	
ELN2022 risk, n (%)					
favorable	51 (44.3)	70 (56.9)	46 (39.3)	53 (42.4)	0.008
intermediate	30 (26.1)	36 (29.3)	52 (44.4)	53 (42.4)	0.010
adverse	7 (6.1)	3 (2.4)	3 (2.6)	6 (4.8)	0.442
missing	27 (23.5)	14 (11.4)	16 (13.7)	13 (10.4)	
Allo HSCT in first CR, n (%)					0.629
Yes	16 (13.9)	13 (10.6)	19 (16.2)	18 (14.4)	
No	99 (86.1)	110 (89.4)	98 (83.8)	107 (85.6)	
missing	0	0	0	0	
Allo HSCT as salvage therapy, n (%)					0.573
Yes	18 (14.3)	25 (18.6)	17 (15.2)	18 (18.4)	
No	97 (73.1)	98 (73.4)	100 (72.6)	107 (73.7)	
missing	0	0	0	0	
Laboratory, median (IQR)					
WBC (10^9^/l)	38.6 (11.0–79.1)	40.1 (17.7–80.1)	36.3 (18.0–66.8)	38.6 (10.9–74.0)	0.7857
HB (mmol/l)	6.3 (5.2–7.6)	6.3 (5.2–7.3)	5.8 (5.2–7.0)	5.9 (5.1–6.9)	0.2230
PLT (10^9^/l)	48 (27–90)	77.5 (50–122)	63 (35–118)	60 (32–109)	0.0018
PBB (%)	67 (37–89)	20 (6–63)	36 (10–65.5)	56 (21–80)	0.0001
BMB (%)	79 (65–88.5)	70 (54.5–83)	70 (49–80)	69.5 (53.5–82.5)	0.0009

Abbrevi: acute myeloid leukemia (AML), secondary AML (sAML), therapy-associated AML (tAML), allogeneic (allo), bone marrow blasts (BMB), hemoglobin (HB), hematopoietic stem cell transplantation (HSCT), interquartile range (IQR), number (n/N), peripheral blood blasts (PBB), platelet count (PLT), white blood cell count (WBC), 95%-confidence interval (95%-CI). Boldface indicates statistical significance (*p* < 0.05)

On the molecular level, *PRDM16*^High^ was associated with higher mutations rates in genes such as *FLT3*-ITD (51% vs 37%; *p* = 0.0258), *DNMT3A* (57% vs 22%; *p* < 0.0001) and *NRAS* (15% vs 5.6%; *p* = 0.014), compared to *PRDM16*^Low^ (Fig. 1C and D). In contrast, *PRDM16*^Low^ correlated with significantly higher frequencies of genomic alterations in *TET2* (30% vs 10%; *p* = 0.0001), *IDH1* (26% vs 10%; *p* = 0.0008), *IDH2* (37% vs 5.5%; *p* < 0.0001), *CEBPA* (13% vs 2.3%; *p* = 0.0019), and *SRSF2* (17% vs 0.7%; *p* < 0.0001), compared to *PRDM16*^High^. Generally, there was a notable trend of increasing rates of mutations interfering with active DNA-demethylation (*TET2*; *IDH1*/*2*) with decreasing *PRDM16* expression: *PRDM16*^High^ (25%), *PRDM16*^Int−II^ (34%), *PRDM16*^Int−I^ (54%), *PRDM16*^Low^ (88%) (Fig. 1C). Further analysis revealed that the *IDH1* R132C and *IDH2* R140Q variants were specifically associated with *PRDM16*^Low^, while no such association was found for the *IDH1* R132H mutation (Fig. 1E and F). Conversely, *PRDM16*^High^ was particularly associated with *DNMT3A* hotspot mutations at codon R882, but not with other *DNMT3A* variants (other *DNMT3A* missense and nonsense mutations; Fig. 1E). Additionally, the integration of mutational profiles across *PRDM16* expression levels revealed that *PRDM16* expression was lowest in AML patients with *TET2*/*IDH1*/*2* alterations and concurrent *DNMT3A*-wildtype status (Fig. 1G).

### Impact of PRDM16 expression levels on clinical outcome

**Fig. 2:**
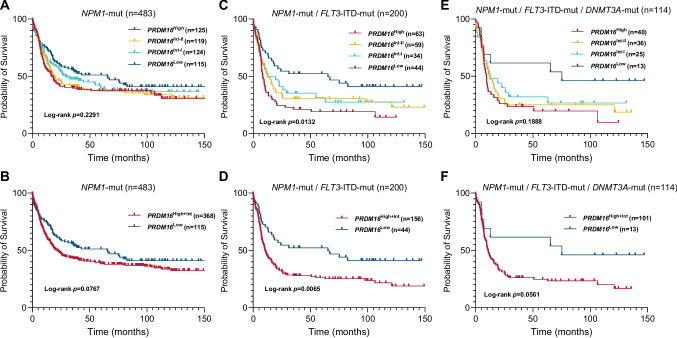
Kaplan–Meier analysis showing the probability of overall survival (OS) for different *PRDM16* expression levels (coded by color) in **(A/B)** the unselected cohort of *NPM1*-mutant AML patients (n = 483), **(C/D)** in *NPM1*-mutant/*FLT3*-ITD-mutant AML subgroup (n = 200), and **(E/F)** in the triple-mutant *NPM1*/*FLT3*-ITD/*DNMT3A* genotype (n = 114). For visualization and statistical power, *PRDM16*^High^ and *PRDM16*^Intermediate^ groups were combined in the lower panels (see Methods)

With respect to clinical outcome, *PRDM16* expression was not associated with overall survival in unselected *NPM1*-mutant AML patients in univariable analysis (Fig. 2A and B; Table 2). Similarly, high *PRDM16* expression was not an independent prognostic factor for EFS, RFS, and OS in multivariable analysis, adjusting for patient’s age at diagnosis, ELN2022 risk categories and the triple-mutant *NPM1*/*DNMT3A*/*FLT3*-ITD genotype (Table S1). Interestingly, *PRDM16*^Low^ was associated with favorable survival (median OS; 65.3 vs 10.4 months; *p* = 0.0065; Fig. 2C and D) in a subgroup analysis within the *NPM1*/*FLT3*-ITD double-mutant genotype (n = 200), compared to AML patients with *PRDM16*^Int/High^. To further examine whether this association depends on *FLT3*-ITD burden, patients were stratified according to the median *FLT3*-ITD variant allele frequency (VAF, 37.7%). The favorable prognostic impact of low *PRDM16* expression remained consistent across both *FLT3*-ITD-low and *FLT3*-ITD-high groups (Supplementary Figure S1A–B), indicating that this effect is independent of *FLT3*-ITD allelic ratio. Likewise, multivariable analysis within the ELN2022 intermediate-risk group, showed that *PRDM16*^Low^ was an independent prognostic factor for longer survival (hazard ratio [95%-CI] 0.467 [0.270–0.807]; *p* = 0.006) in this setting (Table S2). Similarly, there was a trend for even longer OS (median OS; 75.2 vs 11 months; *p* = 0.0561) for *PRDM16*^Low^ within AML patients with the triple-mutant *NPM1*/*DNMT3A*/*FLT3*-ITD (n = 114), but did not reach statistical significance due to the overall low number of *PRDM16*^Low^ AML patients (n = 13) in this specific subgroup (Fig. 2E and F). No impact of *PRDM16* expression levels on clinical outcome was observed in AMLs with the favorable *NPM1*-mutant/*FLT3*-ITD-negative genotype (n = 283) (Figure S2). In addition, no significant association was observed for the relative abundance of the short *PRDM16* isoform with *PRDM16* expression levels or clinical outcome (Figure S3).

**Table 2:** Clinical outcome with respect to *the PRDM16* expression status

Outcome	*PRDM16*^Low^	*PRDM16*^Int−I^	*PRDM16*^Int−II^	*PRDM16*^High^
CR, n (%)	94/115 (81.7)	102/123 (82.9)	95/117 (81.2)	105/125 (84.0)
CR, OR [95%-CI]	1.0	1.08 [0.56–2.11]	0.96 [0.50–1.87]	1.17 [0.60–2.30]
*p* (categorical)		0.810	0.915	0.642
EFS, median (months)	16.7	14.2	9.5	8.2
EFS, HR [95%-CI]	1.0	1.07 [0.78–1.47]	1.18 [0.87–1.62]	1.13 [0.83–1.55]
*p* (categorical)		0.66	0.29	0.43
RFS, median (months)	32.9	23.3	14.9	13.1
RFS, HR [95%-CI]	1.0	1.17 [0.82–1.67]	1.29 [0.90–1.86]	1.26 [0.90–1.86]
*p* (categorical)		0.40	0.16	0.21
OS, median (months)	35.6	27.3	17.7	15.9
OS, HR [95%-CI]	1.0	1.15 [0.82–1.62]	1.33 [0.95–1.86]	1.36 [0.98–1.90]
*p* (categorical)		0.42	0.10	0.07

The variable *PRDM16* was categorized into four levels (low to high). To avoid perfect multicollinearity in the regression analysis, the category *PRDM16*^Low^ was set as the reference group. Abbreviations: Complete remission (CR), event-free survival (EFS), overall survival (OS), relapse-free-survival (RFS), 95%-confidence interval (95%-CI), odds ratio (OR), hazard ratio (HR). Boldface indicates statistical significance (*p* < 0.05)

### PRDM16 promoter methylation

**Fig. 3:**
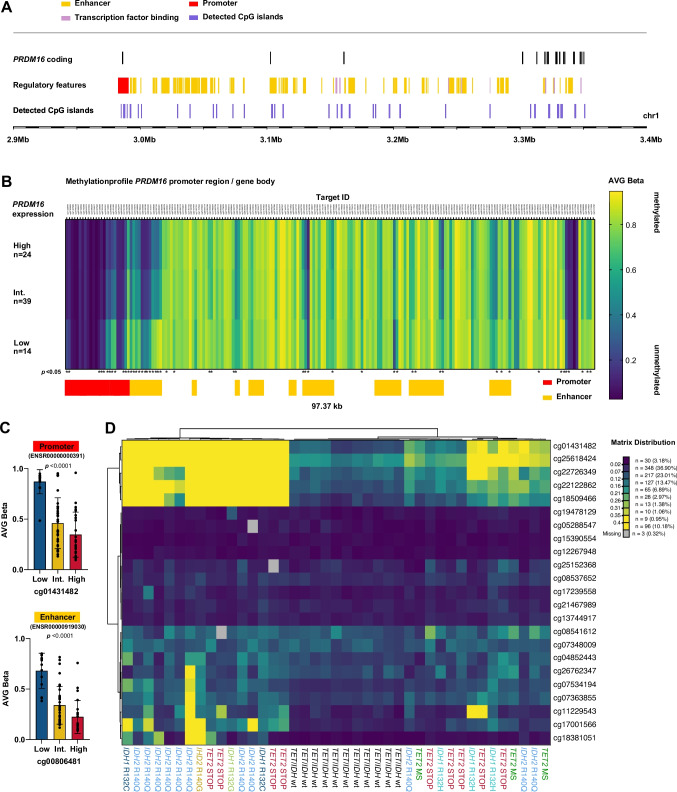
*PRDM16* promoter methylation. **(A)** Analyzed region of the *PRDM16* promoter region and gene body, regulatory sites (coded by color), and detected CpG region. **(B)** AVG-beta values of CpG sites at the *PRDM16* promoter and enhancer regions for different *PRDM16* expression levels: *PRDM16*^Low^, *PRDM16*^Int(Int−I+Int−II)^, and *PRDM16*^High^. **(C)** Comparison of differentially methylated CpG positions (cg01431482 and cg00806481) at *PRDM16* regulatory sites (mean with standard deviation). **(D)** Cluster analysis using Ward algorithm and Euclidean distance between AVG-beta values at the *PRDM16* promoter and the mutational status of *TET2*/*IDH1*/*2*

We detected 31 CpG islands at chromosomal position 1:2,984,445–3,354,467, covering the *PRDM16* promoter region and gene body (exons 1–16) (Fig. 3A). Out of 655 CpG positions analyzed, 128 (19.5%) were differentially methylated across *PRDM16* expression levels. As detailed in table S4, differentially methylated sites included 11/23 (47.8%) CpG positions of the *PRDM16* promoter region (ENSR00000000391; chr1:2,982,601–2991001; hg19) (Fig. 3B and C). In addition, we found differentially methylated loci at various regulatory regions, including 23 enhancer sites and 2 transcription factor-binding sites (Fig. 3B and C; Table S4). The statistically different CpGs in the analyzed regulatory regions were consistently hypermethylated in *PRDM16*^Low^ AMLs, compared to *PRDM16*^Int^ or *PRDM16*^High^ status. Only two CpG position (cg14200569, cg11837181; Table S4) showed a lower methylation level in *PRDM16*^Low^ samples. A more detailed analysis of methylation levels within the *PRDM16* promoter region, further showed a higher degree of methylation in samples harboring the *IDH1* R132C and *IDH2* R140Q variants, and terminating mutations of the *TET2* gene, as compared to other *IDH1*/*IDH2* or *TET2* missense mutations (Fig. 3D). Furthermore, cluster analysis of genomic alterations using methylation data identified a discrete cluster of *PRDM16* promoter hypomethylated AMLs in patients with *TET*/*IDH*-wildtype status (Fig. 3D).

## Discussion

In this study, we analyzed molecular and clinical associations of *PRDM16* expression in a large cohort of *NPM1*-mutant AML patients. Consistent with previous findings, we observed an association of *PRDM16* overexpression with an adverse molecular profile, particularly with a high frequency of *FLT3*-ITD and *DNMT3A* mutations, and a lower ratio of *CEBPA* mutations [15, 17]. This molecular subtype is often implicated in the maintenance of leukemic stem cells, which are known to contribute to chemotherapy resistance and relapse [28]. Therefore, an overall trend for adverse outcome in *NPM1*-mutant AMLs with *PRDM16* overexpression may be an epiphenomenon, reflecting the underlying genetic features rather than an independent driver of AML pathogenesis. Likewise, we did not observe a significant impact of *PRDM16* expression levels in AMLs with a favorable (*NPM1*-mut; *FLT3*-ITD-negative) molecular profile. Interestingly, in AMLs harboring *FLT3*-ITD (and *DNMT3A*) mutations, low *PRDM16* expression correlated with a better prognosis in our study, which aligns with previous reports on a prognostic stratification of *FLT3*-ITD positive AMLs based on the *PRDM16* expression [23]. This supports a putative anti-leukemic effect of reduced *PRDM16* transcription [25] and/or reflects a more favorable epigenetic and metabolic environment that limits leukemic progression, particularly in intermediate and high-risk populations. Similarly, previous studies reported on a prognostic value of *PRDM16* expression in AML with intermediate-risk cytogenetics and normal karyotype [15, 16], which suggests that *PRDM16* may play different roles depending on the broader molecular context. Although *PRDM16* isoforms were shown to differentially regulate normal and leukemic hematopoiesis, with especially the short isoform contributing to hematopoietic stem cells transformation and induction of AML in murine models [19–22], we did not see a differential impact of *PRDM16* isoforms on outcome under clinical conditions in our analysis.

The CpG profiling performed suggests that the methylation status of the *PRDM16* promoter plays a key role in regulating its expression in AML. Likewise, an association of *PRDM16* promoter methylation with expression levels was recently reported in adipose tissue [29], colorectal cancer [30] and astrocytoma cells [31]. We here show, that mutations in genes such as *TET2*, *IDH1/2* as well as *DNMT3A* are directly associated with the epigenetic regulation of *PRDM16* expression. Generally, mutations in *IDH1* and *IDH2* result in the accumulation of the oncometabolite 2-hydroxyglutarate (2-HG), which inhibits the activity of TET enzymes, leading to a hypermethylated genome [32]. In our study, specifically the *IDH1* R132C and *IDH2* R140Q variants were associated with *PRDM16* promoter hypermethylation and gene silencing, pointing at a particular role of these alterations for active DNA demethylation at *PRDM16*. Similarly, terminating lesions in *TET2*, which encodes a DNA demethylase involved in hypermethylation at key regulatory enhancers [33], further contributed to the methylation-driven silencing of *PRDM16*. In addition, mutations of *SRSF2* (at residue P95), which are linked to changes in global mRNA splicing [34], were almost exclusively detected in AMLs with low *PRDM16* expression in our cohort. Interestingly, *SRSF2* P95H mutations alter splicing and expression of various genes critical for early stem cell differentiation and were found to be associated with *PRDM16* downregulation in MDS [35]. Collectively, our findings point at a complex interplay by specific genetic mutations and epigenetic regulation that determine *PRDM16* promoter methylation and expression. Further research is required to validate these findings and to explore the potential for therapies targeting DNA methylation (i.e. by microRNAs) that can potentially modulate *PRDM16* expression or its regulatory mechanisms in AML. For example, miR-101 a tumor suppressor in glioma, was shown to reverse hypomethylation of the *PRDM16* promoter in astrocytoma cells and directly inhibit *PRDM16* expression [31]. Beside the potential as therapeutic target, *PRDM16* expression might be a more refined prognostic tool in *NPM1*-mutated AML, complementing existing risk stratification systems that rely on genetic abnormalities such as *FLT3*-ITD. Ongoing research is needed to better understand the molecular mechanisms underlying these associations and to determine whether *PRDM16* could serve as a predictive biomarker for other specific subtypes of AML. As a limitation, the retrospective nature of our study may have introduced unrecognized selection or temporal biases. However, the consistent treatment backbone and uniform diagnostic procedures across all Study Alliance Leukemia (SAL) protocols likely reduce such confounding effects. Furthermore, although our analysis demonstrates that the prognostic effect of *PRDM16* expression is independent of *FLT3*-ITD variant allele frequency, information on *FLT3*-ITD insertion length and integration site was not consistently available and could therefore not be evaluated in this study.

## Supplementary Information

Below is the link to the electronic supplementary material.

Supplementary file1 (DOCX 253 KB)

## Data Availability

Analyzed datasets are available upon reasonable request from the corresponding author via e-mail.

## Declarations

### Clinical Trial Registration

All patients were treated with intensive regimens in the following clinical trials: AML96 (NCT00180115), AML2003 (NCT00180102), AML60 + (NCT00180167), and SORAML (NCT00893373) or were enrolled in the German Study Alliance Leukemia (SAL)’s AML registry (NCT03188874).

### Permission to reproduce material from other sources

I confirm that no material requiring permission to reproduce from other sources has been included in the paper.
