# Supplementary material for: PRDM16 expression is an independent prognostic factor in AML with the double-mutant NPM1/FLT3-ITD genotype
Source: Ann Hematol. 2026 Jan 22;105(2):49. doi: 10.1007/s00277-026-06767-x (PMC12823675; doi:10.1007/s00277-026-06767-x)
Supplement: Supplementary file 1 — Supplementary file1 (DOCX 253 KB) [file 277_2026_6767_MOESM1_ESM.docx]

**Supplementary Data:** *PRDM16* expression is an independent prognostic factor in AML with the double-mutant *NPM1*/*FLT3*-ITD genotype

| **Event-free survival** | **HR [95%-CI]** | ***p*** |
| --- | --- | --- |
| *PRDM16*^HIGH^ | 1.08 [0.88-1.33] | 0.427 |
| m*NPM1*/m*DNMT3A*/*FLT3*-ITD | 1.01 [0.83-1.24] | 0.913 |
| age | 1.02 [1.02-1.03] | **<0.001** |
| ELN2022 favorable risk | 0.52 [0.42-0.65] | **<0.001** |
| ELN2022 intermediate risk | 0.94 [0.75-1.17] | 0.563 |
| ELN2022 adverse risk | 1.44 [1.16-1.78] | **0.001** |
| **Relapse-free survival** | **HR [95%-CI]** | ***p*** |
| *PRDM16*^HIGH^ | 0.90 [0.71-1.14] | 0.377 |
| m*NPM1*/m*DNMT3A*/*FLT3*-ITD | 1.15 [0.91-1.47] | 0.241 |
| age | 1.02 [1.02-1.03] | **<0.001** |
| ELN2022 favorable risk | 0.59 [0.44-0.79] | **<0.001** |
| ELN2022 intermediate risk | 0.98 [0.73-1.32] | 0.899 |
| ELN2022 adverse risk | 1.26 [0.93-1.70] | 0.139 |
| **Overall survival** | **HR [95%-CI]** | ***p*** |
| *PRDM16*^HIGH^ | 1.10 [0.89-1.37] | 0.373 |
| m*NPM1*/m*DNMT3A*/*FLT3*-ITD | 0.94 [0.75-1.17] | 0.570 |
| age | 1.03 [1.03-1.04] | **<0.001** |
| ELN2022 favorable risk | 0.55 [0.43-0.70] | **<0.001** |
| ELN2022 intermediate risk | 1.00 [0.78-1.27] | 0.979 |
| ELN2022 adverse risk | 1.46 [1.16-1.85] | **0.001** |

**Table S1 Summary of patient outcome with respect to *PRDM16*^HIGH^ status in multivariable analyses.** Square brackets show 95%-confidence intervals. Boldface indicates statistical significance (p<0.05). Abbreviations: hazard ratio (HR).

| **Overall survival in ELN2022 intermediate risk** | **HR [95%-CI]** | ***p*** |
| --- | --- | --- |
| *PRDM16*^Low^ | 0.46 [0.27-0.81] | **0.006** |
| age | 1.03 [1.01-1.05] | **<0.001** |
| Allo HSCT in first CR | 0.66 [0.36-1.21] | 0.181 |
| Allo HSCT as salvage therapy | 0.92 [0.54-1.54] | 0.759 |

**Table S2 Summary of patient outcome with respect to *PRDM16*^Low^ status in ELN2022 intermediate risk in multivariable analyses.** Square brackets show 95%-confidence intervals. Boldface indicates statistical significance (p<0.05). Abbreviations: hazard ratio (HR), Allogeneic hematopoietic stem cell transplantation (Allo HSCT).

| **Variable** | ***PRDM16*^Low^** | ***PRDM16*^Int-I^** | ***PRDM16*^Int-II^** | ***PRDM16*^High^** |
| --- | --- | --- | --- | --- |
| *NPM1*-mut, n/N (%) | 125/503 (24.9) | 127/503 (25.2) | 125/503 (24.9) | 126/503 (25.0) |
| *NPM1*-mut, *FLT3*-ITD-mut, n/N (%) | 46/205 (22.4) | 34/205 (16.6) | 61/205 (29.8) | 64/205 (31.2) |
| *NPM1*-mut, *FLT3*-ITD-mut, *DNMT3A*-mut, n/N (%) | 14/117 (12.0) | 25/117 (21.4) | 37/117 (31.6) | 41/117 (35.0) |
| *NPM1*-mut, *FLT3*-ITD-negative, n/N (%) | 79/298 (26.5) | 93/298 (31.2) | 64/298 (21.5) | 62/298 (20.8) |

**Table S3 Distribution of patients across *PRDM16* expression quartiles within genetically defined AML subgroups.** Values are given as absolute patient numbers followed by percentages in parentheses. Percentages refer to the total number of patients within each molecular subgroup.

|  |  |  | **AVG_Beta** | | |  |
| --- | --- | --- | --- | --- | --- | --- |
| **TargetID** | **Regulatory Region** | **Position (hg19)** | ***PRDM16*^Low^** | ***PRDM16*^Int^** | ***PRDM16*^High^** | ***p*** |
| cg11229543 | **Promoter** | chr1:2984445 | 0.235829 | 0.105457 | 0.103456 | **0.0011** |
| cg07363855 | **Promoter** | chr1:2984470 | 0.194163 | 0.133217 | 0.120664 | **0.0012** |
| cg17239558 | **Promoter** | chr1:2987332 | 0.082979 | 0.054256 | 0.047194 | **0.0039** |
| cg07534194 | **Promoter** | chr1:2987347 | 0.183687 | 0.114947 | 0.093772 | **0.0001** |
| cg18381051 | **Promoter** | chr1:2987645 | 0.144285 | 0.052599 | 0.031737 | **0.001** |
| cg22122862 | **Promoter** | chr1:2987914 | 0.524695 | 0.296068 | 0.216517 | **<0.0001** |
| cg18509466 | **Promoter** | chr1:2987961 | 0.433235 | 0.234695 | 0.165607 | **<0.0001** |
| cg01431482 | **Promoter** | chr1:2989085 | 0.680028 | 0.338881 | 0.222951 | **<0.0001** |
| cg25618424 | **Promoter** | chr1:2989307 | 0.698410 | 0.386619 | 0.279480 | **<0.0001** |
| cg17001566 | **Promoter** | chr1:2990490 | 0.233777 | 0.133381 | 0.099880 | **0.0001** |
| cg22726349 | **Promoter** | chr1:2990678 | 0.533869 | 0.282280 | 0.239332 | **<0.0001** |
| cg25008795 | **Enhancer** | chr1:2992414 | 0.857474 | 0.760371 | 0.637335 | **0.0001** |
| cg14030836 | **Enhancer** | chr1:2992620 | 0.609002 | 0.355301 | 0.286879 | **<0.0001** |
| cg16372810 | **Enhancer** | chr1:2992640 | 0.774575 | 0.605049 | 0.516662 | **<0.0001** |
| cg17890044 | **Enhancer** | chr1:2994372 | 0.743608 | 0.501435 | 0.357055 | **<0.0001** |
| cg05353666 | **Enhancer** | chr1:2995257 | 0.360835 | 0.142568 | 0.122393 | **0.0004** |
| cg12436196 | **Enhancer** | chr1:2995301 | 0.451475 | 0.184691 | 0.125700 | **<0.0001** |
| cg08262220 | **Enhancer** | chr1:2995506 | 0.412442 | 0.189704 | 0.130945 | **<0.0001** |
| cg11731671 | **Enhancer** | chr1:2995604 | 0.556290 | 0.250488 | 0.189248 | **<0.0001** |
| cg25308086 | **Enhancer** | chr1:2996020 | 0.786769 | 0.516502 | 0.390755 | **<0.0001** |
| cg12297125 | **Enhancer** | chr1:2996522 | 0.803689 | 0.474429 | 0.354459 | **<0.0001** |
| cg00806481 | **Enhancer** | chr1:2996650 | 0.869972 | 0.459385 | 0.345137 | **<0.0001** |
| cg22506548 | **Enhancer** | chr1:2996949 | 0.913491 | 0.598922 | 0.522150 | **<0.0001** |
| cg11837181 | **Enhancer** | chr1:3001002 | 0.613574 | 0.687010 | 0.762512 | **<0.0001** |
| cg06724693 | **Enhancer** | chr1:3010896 | 0.623789 | 0.468793 | 0.439777 | **0.0008** |
| cg05223638 | **Enhancer** | chr1:3036168 | 0.772512 | 0.640064 | 0.543992 | **0.0003** |
| cg12473797 | **Enhancer** | chr1:3036358 | 0.579324 | 0.399059 | 0.330465 | **<0.0001** |
| cg16348158 | **Enhancer** | chr1:3036916 | 0.213594 | 0.179453 | 0.118188 | **0.013** |
| cg14200569 | **Enhancer** | chr1:3051925 | 0.244205 | 0.478640 | 0.405721 | **0.008** |
| cg05117823 | **Enhancer** | chr1:3056152 | 0.776016 | 0.652909 | 0.635668 | **<0.0001** |
| cg19243842 | **Enhancer** | chr1:3058521 | 0.711319 | 0.538885 | 0.429392 | **<0.0001** |
| cg22510139 | **Enhancer** | chr1:3058822 | 0.605471 | 0.378509 | 0.265325 | **<0.0001** |
| cg26222332 | **Enhancer** | chr1:3072643 | 0.667283 | 0.425186 | 0.407921 | **0.0002** |
| cg12096707 | **Enhancer** | chr1:3073714 | 0.790264 | 0.690149 | 0.703079 | **0.002** |
| cg03885431 | **DNA binding** | chr1:3153144 | 0.788826 | 0.729172 | 0.672887 | **0.0236** |
| cg03386715 | **DNA binding** | chr1:3153293 | 0.760211 | 0.542231 | 0.519869 | **0.0004** |

**Table S4 Methylation profile of *PRDM16* regulatory regions.** Differentially methylated regions in *PRDM16* promoter, enhancer and transcription factor-binding sites across *PRDM16* expression levels. Boldface indicates statistical significance (p<0.05).


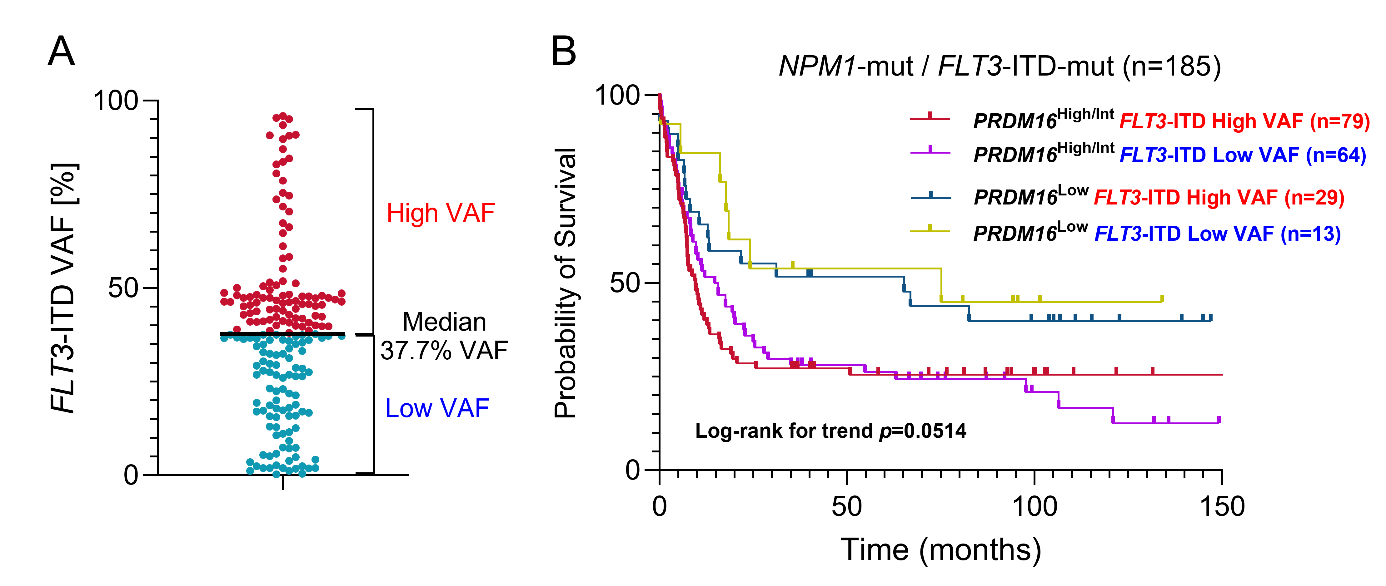


**Figure S1** Association of *PRDM16* expression with clinical outcome according to *FLT3*-ITD variant allele frequency (VAF). (A) Distribution of *FLT3*-ITD VAF values (n = 185) with the median of 37.7 % used as the dichotomization threshold separating *FLT3*-ITD-low (blue) and *FLT3*-ITD-high (red) subgroups. (B) Overall survival of patients with *PRDM16*^Low^ versus *PRDM16*^High/Intermediate^ expression within the *FLT3*-ITD-low and *FLT3*-ITD-high subgroups.


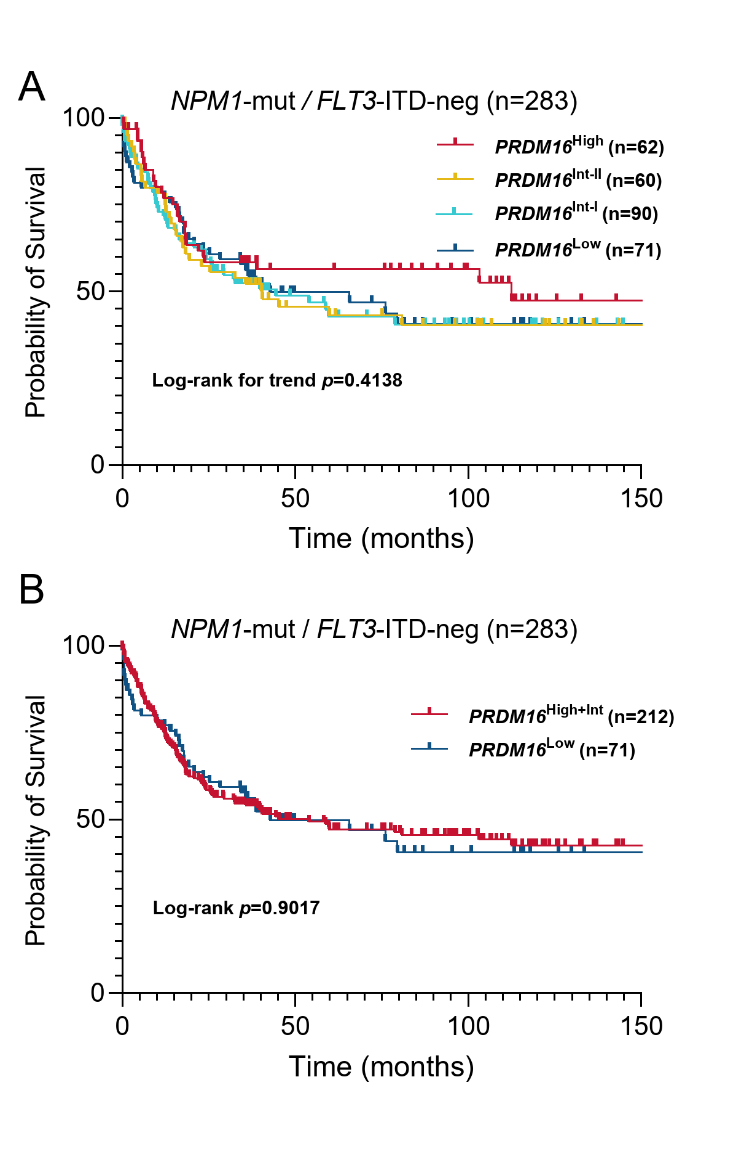


**Figure S2** Kaplan-Meier analysis showing the probability of overall survival (OS) for different *PRDM16* expression levels (coded by color) in *NPM1*-mutant / *FLT3*-ITD-negative AML patients (n=283). For visualization and statistical power, *PRDM16*^High^ and *PRDM16*^Intermediate^ groups were combined in the lower panel (see Methods).


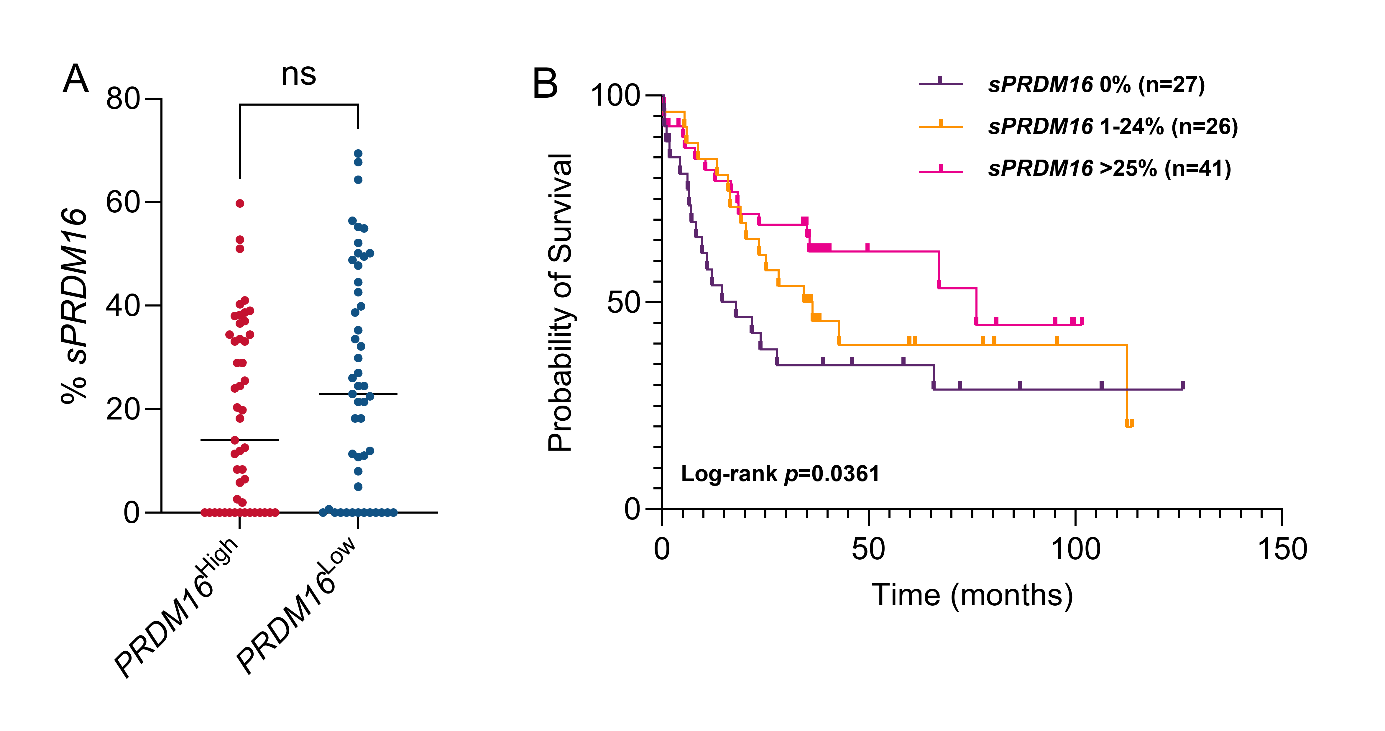


**Figure S3** Association of the short *PRDM16* (*sPRDM16*) isoform with (A) *PRDM16* expression levels and (B) clinical outcome (OS).
